# “Health supply chain personnel: an integral part of the health workforce.”

**DOI:** 10.1186/2052-3211-7-S1-I1

**Published:** 2014-12-17

**Authors:** Giorgio Cometto, Zaheer-Ud-Din Babar, Andrew Brown, Lisa Hedman, James Campbell

**Affiliations:** 1 Global Health Workforce Alliance, World Health Organization, Geneva, Switzerland; 2 School of Pharmacy, Faculty of Medical and Health Sciences University of Auckland, New Zealand; 3 People that Deliver, UNICEF, Copenhagen, Denmark; 4 Essential Medicines and Health Products, World Health Organization, Geneva, Switzerland

## 

Approximately a third of the world population – and about half in the most underdeveloped settings – have been estimated to lack access to essential medicines and diagnostics [1]. Effective supply chains are vital to deliver essential health commodities. In high-income countries the availability of medicines in the public and private sector is taken as a given: quality assurance is managed by robust national regulatory agencies; supply and distribution are increasingly privatized, with performance measured against timeliness and cost. Conversely, in many low- and middle-income countries, stock-outs of essential commodities are commonplace, with a mean availability of core medicines in the public sector ranging from 38.2% in sub-Saharan Africa to 57.7 % in Latin America and the Caribbean [2]. Vulnerability of supply chain functions also increases the potential for the entry of counterfeit and substandard products [3].

While availability of medicines is determined by multiple factors, there is a growing recognition of the need to address human resources requirements for supply chain systems [4]. A systematic review of the global pharmacy workforce revealed a dearth of evidence from low- and middle- income countries [5] . It also underscored several challenges, including inadequate numbers of pharmacists and pharmacy support workforce cadres, issues of maldistribution (across public and private sectors, and urban and rural areas), uneven implementation of education, staff management and retention strategies. Further, this study did not find evidence on the broader range of health logisticians and supply chain managers. Other analyses focused on low- and middle-income settings have highlighted dramatic supply chain workforce shortages, with some countries facing vacancy rates up to 71% for public sector posts that would require accredited pharmaceutical training [6]. This situation is often determined by a combination of insufficient training capacity as well as 100-150% higher wages in the private sector as compared to the public sector.

Some of these problems reflect those affecting human resources for health more broadly. A cross-country analysis of the health workforce conducted in 2013 showed that multi-pronged strategies are required to improve forecasting, planning, education, deployment, retention and performance management of human resources for health [7].Only by addressing these factors in an integrated manner, will it be possible for health systems to improve availability, accessibility, acceptability and quality of the human resources. This is a requirement to accelerate progress towards attaining universal health coverage.

Better health workforce intelligence and data can shape more effective planning, implementation and monitoring of such policies. A stronger evidence base on quantities, geographic distribution, competency frameworks, as well as the labour market conditions that determine the availability and performance of the health supply chain personnel, would similarly be required.

A more effective response to health supply chain workforce challenges therefore requires comprehensive and reliable data on availability, distribution, education curricula, competency frameworks, levels of remuneration, regulatory environment and supporting systems. Dedicated tools exist for assessment of operational and technical capacity in public health supply chain personnel [8], and related analyses have been conducted in some contexts [9,10]. There are also good governance initiatives focusing on legislation, transparency and integrity to reduce corruption and advance the professionalization of the supply chain profession [11]. Both these aspects are important, however existing initiatives have not yet fully captured the need for a leadership environment that promotes excellence and attracts talent, and that explicitly links the health supply chain system with a country’s broader public health goal of promoting equitable access to essential medicines.

In most countries a relative lack of comprehensive data on supply chain personnel (and especially on the administrators, logistics managers, warehouse and transport personnel, clerks and other support cadres) means that critical capacity gaps go unnoticed, and often neglected in national health and human resources policies and strategies. Nevertheless, the supply chain workforce should be fully embedded in the core functions of health workforce management, including the human resources for health information systems, planning and forecasting, performance management [12]. Achieving this integration can be facilitated by an enabling policy and governance framework at the country and regional level.

Some of the required actions to strengthen the health supply chain workforce may be similar to - or be implemented as part of - broader health workforce policies. This includes improving public sector pay and incentives [13]; establishing rural pipelines to education and training to facilitate education and deployment in under-served areas [14]; reforming education strategies to adapt content and modalities of training to current and emerging health system needs [15]; and exploring the potential of greater delegation of tasks to cadres with shorter training [16]. Other interventions may need to be more specific to the supply chain workforce, such as mainstreaming relevant competencies in the pre-service education curricula of health personnel; scaling up training of pharmacists and pharmacy assistants; and professionalizing the personnel in administrative and management positions within the health supply system through more dedicated training (which may also help in countering the increasing burden on the functions of clinical staff). Key skills are particularly required in forecasting of needs, procurement, quality assurance, warehousing and distribution, stock management, with an overarching need for leadership and systems management.

The implementation of conducive supply chain workforce policies may require additional financing commitments or re-allocating available resources. However, considering the enormous levels of wastage associated with inadequate, ineffective and irrational procurement of medicines and other health commodities [17], investments in the health supply chain personnel may represent a strategy to improve the overall efficiency of health systems, and may therefore represent an area worth prioritizing [18].

In a nutshell, health systems throughout the world are progressively broadening their focus to non-communicable diseases, and are attempting to expand effective coverage to under-served populations through equity-focused policies and quality enhancement interventions. The emerging discourse on the Sustainable Development Goals in the context of the post-2015 agenda includes eliminating avoidable maternal and child deaths, controlling epidemic diseases, and explicitly refers to providing “access to affordable essential medicines and vaccines” [19].

Strengthening the supply chain workforce is an essential element of making this vision a reality. This special supplement seeks to expand the evidence base contributing to the 2^nd^ People that Deliver Global Conference on Human Resources for Supply Chain Management (http://www.peoplethatdeliver.org). This event marks the beginning of a second phase of the People that Deliver Initiative, which will place growing emphasis on country-focused action.
